# PTEN Is Required for The Anti-Epileptic Effects of AMPA Receptor Antagonists in Chronic Epileptic Rats

**DOI:** 10.3390/ijms21165643

**Published:** 2020-08-06

**Authors:** Ji-Eun Kim, Hana Park, Ji-Eun Lee, Tae-Hyun Kim, Tae-Cheon Kang

**Affiliations:** 1 Department of Anatomy and Neurobiology, College of Medicine, Hallym University, Chuncheon 24252, Korea; jieunkim@hallym.ac.kr (J.-E.K.); M19050@hallym.ac.kr (H.P.); 20183533@hallym.ac.kr (J.-E.L.); hyun1028@hallym.ac.kr (T.-H.K.); 2 Institute of Epilepsy Research, College of Medicine, Hallym University, Chuncheon 24252, Korea

**Keywords:** AMPA receptor, BpV(pic), GluA, GluR, GYKI 52466, perampanel, seizure

## Abstract

α-Amino-3-hydroxy-5-methyl-4-isoxazolepropionic acid receptor (AMPAR) is one of the ligand-gated ion channels for glutamate, which is an important player in the generation and spread of seizures. The efficacy of AMPAR functionality is regulated by the trafficking, synaptic targeting, and phosphorylation. Paradoxically, AMPAR expression and its phosphorylation level are decreased in the epileptic hippocampus. Therefore, the roles of AMPAR in seizure onset and neuronal hyperexcitability in ictogenesis remain to be elucidated. In the present study, we found that AMPAR antagonists (perampanel and GYKI 52466) decreased glutamate ionotropic receptor AMPA type subunit 1 (GRIA1) surface expression in the epileptic rat hippocampus. They also upregulated phosphatase and tensin homolog deleted on chromosome 10 (PTEN) expression and restored to basal levels the upregulated phosphoinositide 3-kinase (PI3K)/AKT1 phosphorylations. Dipotassium bisperoxovanadium(pic) dihydrate (BpV(pic), a PTEN inhibitor) co-treatment abolished the anti-epileptic effects of perampanel and GYKI 52466. Therefore, our findings suggest that PTEN may be required for the anti-epileptic effects of AMPAR antagonists.

## 1. Introduction

Epilepsy is one of the most common chronic neurological disorders, which is characterized by the presence of spontaneous episodes of abnormal excessive neuronal discharges [1,2]. α-Amino-3-hydroxy-5-methyl-4-isoxazolepropionic acid receptor (AMPAR) is one of the ligand-gated ion channels for glutamate. AMPAR has four subunits (GRIA1-4, also named GluA1-4 or GluR1-4), which compose the tetrameric structures of the receptor. The phosphorylations of GRIA1 subunit at serine (S) residues 831 (S831) and 845 (S845) sites play important roles in governing the single-channel conductance, recruiting new high-conductance-state of AMPAR, and stabilizing its surface expression [3,4,5]. Since AMPAR plays a role in fast postsynaptic depolarization through Na^+^ influx and, to a lesser extent, Ca^2+^, it is involved in seizure initiation, epileptic synchronization, and the spread of seizure activity. Thus, AMPAR is one of the major therapeutic targets in the treatment of epilepsy [4,5]. As compared to the control (normal) hippocampus, however, AMPAR expression and its phosphorylation levels are decreased in the epileptic hippocampus [6,7,8,9]. Therefore, the roles of AMPAR in the onset of seizures and the alterations of neuronal excitability in the epileptic hippocampus remain to be elucidated.

Phosphatase and tensin homolog deleted on chromosome 10 (PTEN), classically identified as a tumor suppressor, negatively regulates the phosphoinositide 3-kinase (PI3K)/AKT1 signaling pathway by dephosphorylating phosphatidylinositol 3,4,5-trisphosphate (PIP3) to phosphatidylinositol 4,5-bisphosphate (PIP2). PI3K maintains membrane AMPAR clustering and its surface expression, which strongly modulates the synaptic strength and memory formation [10,11,12]. Furthermore, mutation or inactivation of PTEN contributes to seizure generation [13,14,15,16]. Considering that PTEN negatively contributes to AMPAR trafficking and its surface expression [17], it is plausible that dysfunction of PTEN would generate seizure activity via the increased PI3K-mediated AMPAR trafficking in the epileptic hippocampus, which is less known.

Here, we provide the first compelling evidence that in the epileptic hippocampus nuclear factor-kappa B (NF-κB)-mediated PTEN downregulation activated the PI3K/AKT1 signaling pathway, which led to increased membrane GRIA1/total GRIA1 ratio (Memb./Total GRIA1 ratio). Blockade of AMPAR by perampanel or GYKI 52,466 reduced surface GRIA1 expression and spontaneous seizure activity, accompanied by restoring PTEN-mediated PI3K/AKT1 inhibition. Dipotassium bisperoxovanadium(pic) dihydrate (BpV(pic), a PTEN inhibitor) abrogated the anti-epileptic effects of perampanel or GYKI 52466. Therefore, these findings suggest that PTEN may be required for the anti-epileptic effects of AMPAR antagonists.

## 2. Results

### 2.1. AMPAR Inhibition Reduces Surface GRIA1 Expression in the Epileptic Hippocampus

**Figure 1:**
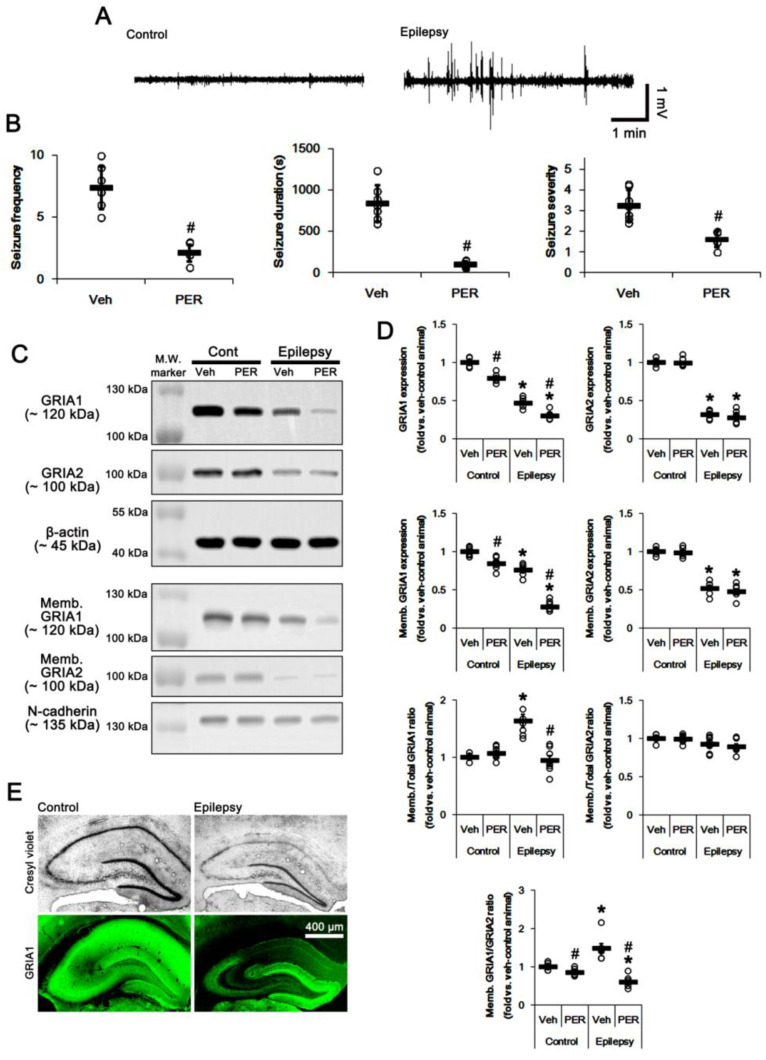
The effect of perampanel (PER) on spontaneous seizure activity, total glutamate ionotropic receptor AMPA type subunit 1 (GRIA1) and membrane GRIA1 expression in control (cont) and epileptic rats. (**A**) Representative electroencephalogram (EEG) traces obtained from control and epileptic rats. (**B**) Quantitative values of seizure frequency, total seizure duration, and seizure severity during 2 h of recording a day. Open circles indicate each individual value. Horizontal bars indicate mean value. Error bars indicate SD (* p < 0.05 vs. vehicle (Veh)-treated animals; Mann–Whitney U-test for seizure frequency and seizure severity; Student’s *t*-test for seizure duration; *n* = 7, respectively). (C) Representative images for Western blot of GRIA1, GRIA2, membrane GRIA1, and membrane GRIA2 levels in the hippocampal tissues. (D) Quantifications of GRIA1, GRIA2, membrane GRIA1, and membrane GRIA2 levels in the hippocampal tissues. Open circles indicate each individual value. Horizontal bars indicate mean value. Error bars indicate SEM (*, # *p* < 0.05 vs. vehicle (Veh)-treated control animal and vehicle-treated animals, respectively; one-way ANOVA with post hoc Bonferroni’s multiple comparison; *n* = 7, respectively). (E) Representative photos of remaining neurons after SE (Cresyl violet) and GRIA1 expression in the control and epileptic rats.

In epileptic rats (6 weeks after SE), the seizure frequency (number of seizures) was 7.4 ± 1.7/recording session (2 h) and the total seizure duration (the overall time spent in convulsive and non-convulsive seizures) on electroencephalograms (EEG) was 845.3 ± 219.2 s. The seizure severity (behavioral seizure core) was 3.3 ± 0.8 (Figure 1A,B). Consistent with previous reports [6,8,9,18], total GRIA1 and GRIA2 expression levels were significantly reduced in the epileptic hippocampus at 7 weeks after SE (*p* < 0.05 vs. control animals, one-way ANOVA with post hoc Bonferroni’s multiple comparison, *n* = 7, respectively; Figure 1C,D and Figure S1). Immunohistochemical study revealed that the decreased GRIA1 expression in the CA1-3 regions, but not the dentate gyrus, resulted in the reduced total GRIA1 expression (Figure 1E). Similar to the amount of total GRIA1, the epileptic hippocampus showed reduced GRIA1 expression in membrane fraction (*p* < 0.05 vs. control animals, one-way ANOVA with post hoc Bonferroni’s multiple comparison, *n* = 7, respectively; Figure 1C,D and Figure S1). However, the membrane GRIA1/total GRIA1 ratio (Memb./Total GRIA1 ratio) in the epileptic hippocampus was ≈ 1.5-fold higher than that in control hippocampus (*p* < 0.05 vs. control animals, one-way ANOVA with post hoc Bonferroni’s multiple comparison, *n* = 7, respectively; Figure 1C,D). However, Memb./Total GRIA2 ratio was similar to that in control animals (Figure 1C,D). Thus, membrane GRIA1/GRIA2 ratio (Memb. GRIA1/GRIA2 ratio) was 1.49-fold higher than that in control animals (*p* < 0.05 vs. control animals, one-way ANOVA with post hoc Bonferroni’s multiple comparison, *n* = 7, respectively; Figure 1C,D). These findings indicate that the increased surface GRIA1, but not GRIA2, expression/trafficking may be involved in the ictogenesis in epileptic rats.

To explore the role of AMPAR hyperactivation in spontaneous seizure generations, we applied perampanel, a non-competitive antagonist of AMPAR. Perampanel treatment significantly reduced seizure activity (*n* = 7 out of 11): the seizure frequency was 2.1 ± 0.7/recording session and the total seizure duration was 100.7 ± 38.7 s (*p* < 0.05 vs. vehicle *n* = 7, Mann–Whitney U-test and Student’s *t*-test, respectively; Figure 1A,B). The seizure severity was 1.6 ± 0.4 (*p* < 0.05 vs. vehicle, Mann–Whitney U-test, *n* = 7, respectively; Figure 1A,B). Four rats in the perampanel-treated group were identified as non-responders to perampanel, whose seizure frequency and seizure duration were unaffected by perampanel. Only responders to perampanel were used for the data analysis and the biochemical study. As compared to vehicle, perampanel reduced total- and membrane GRIA1 expressions to 0.79- and 0.85-fold of vehicle level in control animals, respectively (*p* < 0.05 vs. vehicle, one-way ANOVA with post hoc Bonferroni’s multiple comparison, *n* = 7, respectively; Figure 1C,D and Figure S1). Perampanel did not influence total- and surface expression of GRIA2 in control animals (Figure 1C,D and Figure S1). Thus, Memb. GRIA1/GRIA2 ratio was reduced to 0.88-fold of vehicle level in control animals (*p* < 0.05 vs. vehicle, one-way ANOVA with post hoc Bonferroni’s multiple comparison, *n* = 7, respectively; Figure 1C,D). In epileptic rats, perampanel diminished total- and membrane GRIA1 expression to 0.64- and 0.37-fold of vehicle level, respectively (*p* < 0.05 vs. vehicle, one-way ANOVA with post hoc Bonferroni’s multiple comparison, *n* = 7, respectively; Figure 1C,D). Perampanel also decreased Memb./Total GRIA1 ratio to control level (*p* < 0.05 vs. vehicle, one-way ANOVA with post hoc Bonferroni’s multiple comparison, *n* = 7, respectively; Figure 1C,D). However, perampanel did not affect total- and membrane GRIA2 expression in the epileptic hippocampus. Perampanel decreased Memb. GRIA1/GRIA2 ratio to 0.4-fold of vehicle level (*p* < 0.05 vs. vehicle, one-way ANOVA with post hoc Bonferroni’s multiple comparison, *n* = 7, respectively; Figure 1C,D). These findings indicate that the anti-epileptic effect of perampanel may be relevant to the reductions in surface GRIA1 expression and Memb. GRIA1/GRIA2 ratio in the epileptic hippocampus.

**Figure 2:**
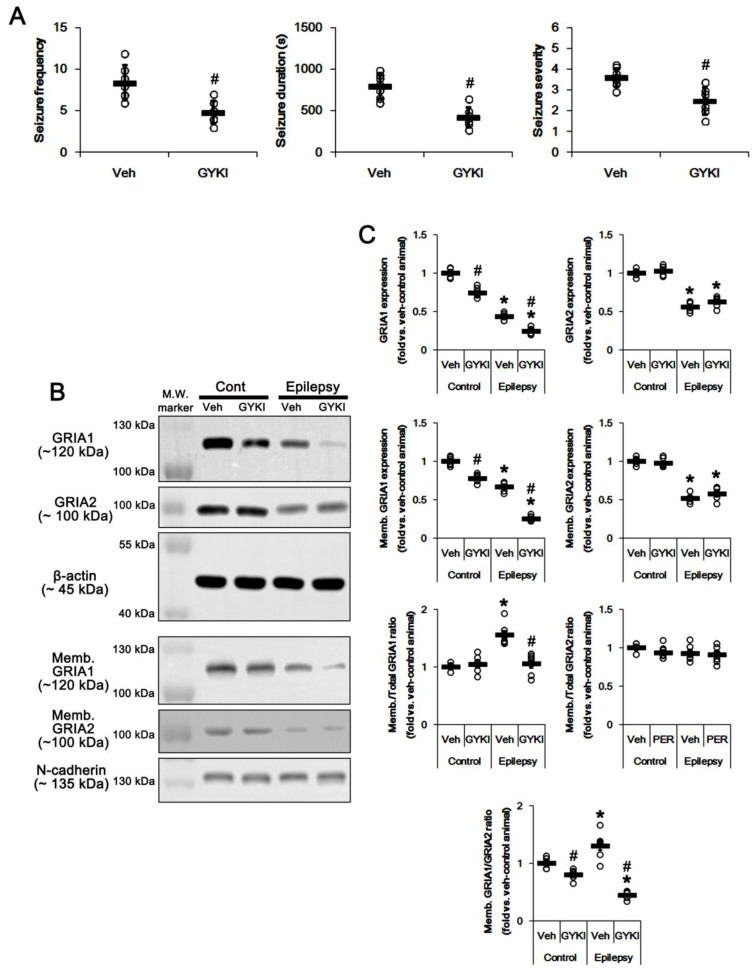
The effect of GYKI 52,466 (GYKI) on spontaneous seizure activity, total GRIA1, and membrane GRIA1 expression in control (cont) and epileptic rats. (**A**) Quantitative values of seizure frequency, total seizure duration, and seizure severity during 2 h of recording a day. Open circles indicate each individual value. Horizontal bars indicate mean value. Error bars indicate SD (* p < 0.05 vs. vehicle (Veh)-treated animals; Mann–Whitney U-test for seizure frequency and seizure severity; Student’s *t*-test for seizure duration; *n* = 7, respectively). (B) Representative images for Western blot of GRIA1, GRIA2, membrane GRIA1, and membrane GRIA2 levels in the hippocampal tissues. (C) Quantifications of GRIA1, GRIA2, membrane GRIA1, and membrane GRIA2 levels in the hippocampal tissues. Open circles indicate each individual value. Horizontal bars indicate mean value. Error bars indicate SEM (*, # *p* < 0.05 vs. vehicle (Veh)-treated control animal and vehicle-treated animals, respectively; one-way ANOVA with post hoc Bonferroni’s multiple comparison; *n* = 7, respectively).

To confirm that the blockade of AMPAR would affect GRIA1 surface expression, we also applied GYKI 52466, another allosteric AMPAR inhibitor (a non-competitive AMPAR antagonist). Similar to perampanel, GYKI 52,466 significantly attenuated seizure activity (*n* = 7 out of 12): the seizure frequency was 4.6 ± 1.3/recording session (*p* < 0.05 vs. vehicle, Mann–Whitney U-test, *n* = 7, respectively; Figure 2A). The total seizure duration was 428 ± 119.3 s (*p* < 0.05 vs. vehicle, Student’s *t*-test, *n* = 7, respectively; Figure 2A). The seizure severity was 2.5 ± 0.6 (*p* < 0.05 vs. vehicle, Mann–Whitney U-test, *n* = 7, respectively; Figure 2A). Non-responders to GYKI 52,466 (*n* = 5) were not used for the biochemical study. GYKI 52,466 reduced total- and membrane GRIA1 expression to 0.75- and 0.78-fold of vehicle level in control animals, respectively (*p* < 0.05 vs. vehicle, *n* = 7, respectively; Figure 2B,C and Figure S2). GYKI 52,466 did not influence Memb./Total GRIA1 ratio in control animals (Figure 2B,C). Since GYKI 52,466 did not affect total- and surface expression of GRIA2 (Figure 2B,C and Figure S2), it diminished Memb. GRIA1/GRIA2 ratio to 0.8-fold of vehicle level in control animals (*p* < 0.05 vs. vehicle, one-way ANOVA with post hoc Bonferroni’s multiple comparison, *n* = 7, respectively; Figure 2B,C). In epileptic rats, GYKI 52,466 diminished total- and membrane GRIA1 expression to 0.56- and 0.38-fold of vehicle level, respectively (*p* < 0.05 vs. vehicle, one-way ANOVA with post hoc Bonferroni’s multiple comparison, *n* = 7, respectively; Figure 2B,C and Figure S2). GYKI 52,466 decreased Memb./Total GRIA1 ratio to control level (*p* < 0.05 vs. vehicle, one-way ANOVA with post hoc Bonferroni’s multiple comparison, *n* = 7, respectively; Figure 2B,C). GYKI 52,466 did not affect total- and membrane GRIA2 expression in the epileptic hippocampus. Thus, GYKI 52,466 reduced Memb. GRIA1/GRIA2 ratio to 0.34-fold of vehicle level in the epileptic hippocampus (*p* < 0.05 vs. vehicle, one-way ANOVA with post hoc Bonferroni’s multiple comparison, *n* = 7, respectively; Figure 2B,C). These findings indicate that the blockade of AMPAR may abrogate GRIA1 trafficking.

### 2.2. Blockade of AMPAR Abolishes the Upregulation of PI3K/AKT1 Activities in The Epileptic Hippocampus

**Figure 3:**
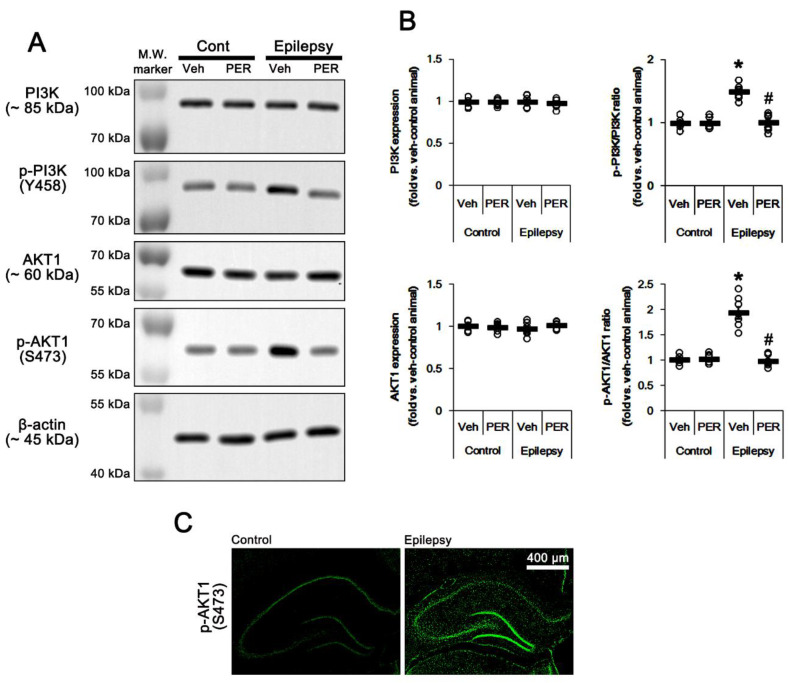
The effect of perampanel (PER) on phosphoinositide 3-kinase (PI3K) and AKT1 expressions and their phosphorylations in control (cont) and epileptic rats. (**A**) Representative images for Western blot of PI3K, phospho (p)-PI3K, AKT1, and p-AKT1 level in the hippocampal tissues. (**B**) Quantifications of PI3K, phospho (p)-PI3K, AKT1, and p-AKT1 level in the hippocampal tissues. Open circles indicate each individual value. Horizontal bars indicate mean value. Error bars indicate SEM (*, # *p* < 0.05 vs. vehicle (Veh)-treated control animal and vehicle-treated animals, respectively; one-way ANOVA with post hoc Bonferroni’s multiple comparison; *n* = 7, respectively). (**C**) Representative photos of p-AKT1 signals in the control and epileptic rats.

**Figure 4:**
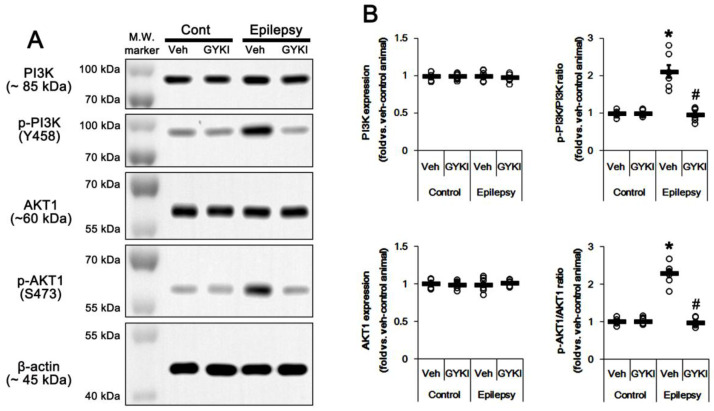
The effect of GYKI 52,466 (GYKI) on PI3K and AKT1 expressions and their phosphorylations in control (cont) and epileptic rats. (A) Representative images for Western blot of PI3K, phospho (p)-PI3K, AKT1, and p-AKT1 level in the hippocampal tissues. (B) Quantifications of PI3K, phospho (p)-PI3K, AKT1, and p-AKT1 level in the hippocampal tissues. Open circles indicate each individual value. Horizontal bars indicate mean value. Error bars indicate SEM (*, # *p* < 0.05 vs. vehicle (Veh)-treated control animal and vehicle-treated animals, respectively; one-way ANOVA with post hoc Bonferroni’s multiple comparison; *n* = 7, respectively).

The PI3K/AKT1 signaling pathway plays an important role in AMPAR transmission by regulating membrane AMPAR clustering and its trafficking [10,11,12]. Thus, we investigated whether AMPAR inhibition affects PI3K/AKT1 expressions and their phosphorylations (activities). As compared to control animals, both PI3K Y458 and AKT1 S473 phosphorylation ratio was increased to 1.5- and 1.93-fold of control level in the epileptic hippocampus without changing their expression levels, respectively (*p* < 0.05 vs. control animals, one-way ANOVA with post hoc Bonferroni’s multiple comparison, *n* = 7, respectively; Figure 3A,B and Figure S3). AKT1 S473 phosphorylation level was enhanced in the dentate granule cells, although it was reduced CA1 pyramidal cells (Figure 3C). Perampanel reduced PI3K Y458 and AKT1 S473 phosphorylation ratio to control levels in epileptic rats, but it did not affect the same enzymes in control animals (*p* < 0.05 vs. vehicle, one-way ANOVA with post hoc Bonferroni’s multiple comparison, *n* = 7, respectively; Figure 3A,B and Figure S3). Similar to perampanel, GYKI 52,466 significantly diminished both PI3K Y458 and AKT1 S473 phosphorylation ratio to control level in epileptic rats (*p* < 0.05 vs. vehicle, one-way ANOVA with post hoc Bonferroni’s multiple comparison, *n* = 7, respectively; Figure 4A,B and Figure S4). These findings indicate that the upregulated PI3K and AKT1 activities may be relevant to the increased surface GRIA1 expression in the epileptic hippocampus, which may lead to ictogenesis.

### 2.3. Blockade of AMPAR Attenuates NF-κB-Mediated PTEN Downregulation in the Epileptic Hippocampus

**Figure 5:**
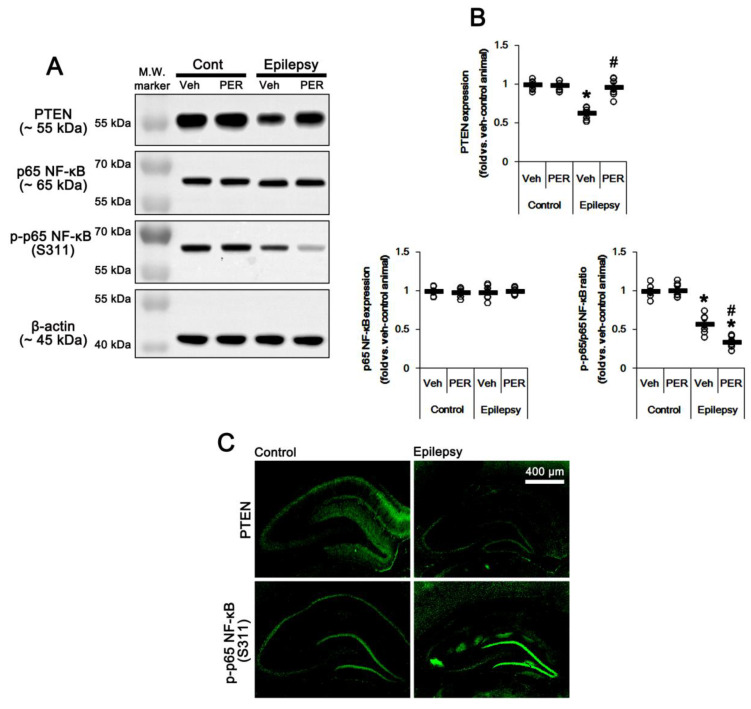
The effect of perampanel (PER) on phosphatase and tensin homolog deleted on chromosome 10 (PTEN) and p65 nuclear factor-kappa B (NF-κB) expression/phosphorylation in control (cont) and epileptic rats. (**A**) Representative images for Western blot of PTEN, p65 NF-κB, and p-p65 NF-κB level in the hippocampal tissues. (**B**) Quantifications of PTEN, p65 NF-κB, and p-p65 NF-κB level in the hippocampal tissues. Open circles indicate each individual value. Horizontal bars indicate mean value. Error bars indicate SEM (*, # *p* < 0.05 vs. vehicle (Veh)-treated control animal and vehicle-treated animals, respectively; one-way ANOVA with post hoc Bonferroni’s multiple comparison; *n* = 7, respectively). (**C**) Representative photos of PTEN and p-p65 NF-κB in the control and epileptic rats.

**Figure 6:**
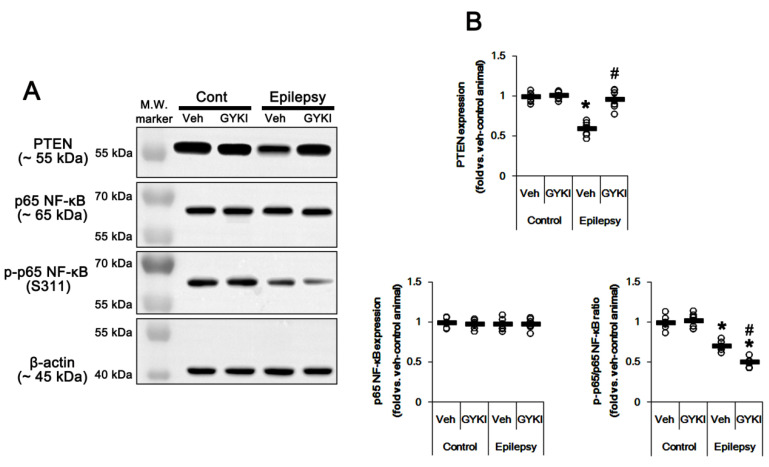
The effect of GYKI 52,466 (GYKI) on PTEN and p65 NF-κB expression/phosphorylation in control (cont) and epileptic rats. (**A**) Representative images for Western blot of PTEN, p65 NF-κB, and p-p65 NF-κB level in the hippocampal tissues. (**B**) Quantifications of PTEN, p65 NF-κB, and p-p65 NF-κB level in the hippocampal tissues. Open circles indicate each individual value. Horizontal bars indicate mean value. Error bars indicate SEM (*, # *p* < 0.05 vs. vehicle (Veh)-treated control animal and vehicle-treated animals, respectively; one-way ANOVA with post hoc Bonferroni’s multiple comparison; *n* = 7, respectively).

Since PTEN is the negative upstream regulator of PI3K/AKT1 pathway [10,11,12], we explored whether PTEN expression and its phosphorylation are altered in the epileptic hippocampus. As compared to control animals, PTEN expression was ≈0.6-fold lower in epileptic rats (*p* < 0.05 vs. control animals, one-way ANOVA with post hoc Bonferroni’s multiple comparison, *n* = 7, respectively; Figure 5A,B and Figure S5). PTEN expression was reduced in dentate granule cells as well as CA1 pyramidal cells (Figure 5C). Perampanel and GYKI 52,466 increased PTEN expression to control level in the epileptic hippocampus (*p* < 0.05 vs. vehicle, *n* = 7, respectively; one-way ANOVA with post hoc Bonferroni’s multiple comparison, Figure 5A,B, Figure 6A,B, Figure S5 and S6). Together with upregulation of PI3K/AKT1 phosphorylation, these findings indicate that AMPAR-mediated spontaneous seizure activity may abolish PTEN activity in the epileptic hippocampus.

AMPAR activation increases NF-κB activity [19,20]. p65-Ser311 NF-κB phosphorylation is a hallmark of NF-κB function to activate NF-κB-dependent transcription [21]. Interestingly, seizure activity elevates p65-Ser311 NF-κB phosphorylation in dentate granule cells, but reduces it in CA1 and CA3 neurons [22,23]. Since NF-κB negatively regulates PTEN expression [24], it is likely that seizure-induced NF-κB activation may decrease PTEN expression in the epileptic hippocampus. As compared to control animals, p65-Ser311 NF-κB phosphorylation ratio was reduced ≈0.6-fold of control animal level in epileptic rats without altering NF-κB expression level (*p* < 0.05 vs. control animals, one-way ANOVA with post hoc Bonferroni’s multiple comparison, *n* = 7, respectively; Figure 5A,B and Figure S5). p65-Ser311 NF-κB phosphorylation was enhanced in dentate granule cells, while it was reduced in CA1-3 pyramidal cells due to neuronal loss (Figure 5C). Perampanel and GYKI 52,466 decreased p65-Ser311 NF-κB phosphorylation ratio to 0.6- and 0.7-fold of vehicle level in the epileptic hippocampus (*p* < 0.05 vs. vehicle, one-way ANOVA with post hoc Bonferroni’s multiple comparison, *n* = 7, respectively; Figure 5A,B, Figure 6A,B, Figure S5 and S6). Therefore, these finding indicate that upregulation of NF-κB activity may diminish PTEN activity in the epileptic hippocampus.

### 2.4. PTEN Inhibitor Abrogates the Anti-Epileptic Effects of Perampanel and GYKI 52,466 in Epileptic Rats

**Figure 7:**
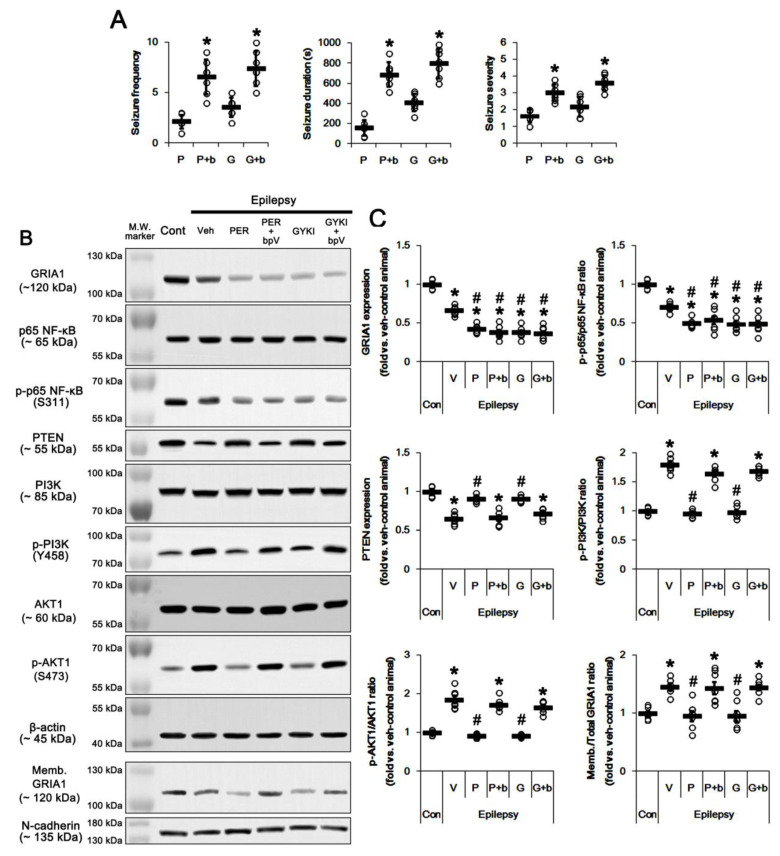
The effect of co-treatment of dipotassium bisperoxovanadium(pic) dihydrate (BpV(pic)) (bpv, b) with perampanel (PER, P) or GYKI 52,466 (GYKI, G) on spontaneous seizure activity, total/membrane GRIA1 expressions, PI3K/AKT1, PTEN, and p65 NF-κB expressions/phosphorylations in control (cont) and epileptic rats. BpV co-treatment abrogates the effects of perampanel or GYKI 52466. (**A**) Quantitative values of seizure frequency, total seizure duration, and seizure severity during 2 h of recording a day. Open circles indicate each individual value. Horizontal bars indicate mean value. Error bars indicate SD (* p < 0.05 vs. perampanel- or GYKI 52466-treated animals; Mann–Whitney U-test for seizure frequency and seizure severity; Student’s *t*-test for seizure duration; *n* = 7, respectively). (B) Representative images for Western blots concerning GRIA1, p65 NF-κB, PTEN, PI3K, and AKT1 expressions/phosphorylations. (C) Quantifications of GRIA1, p65 NF-κB, PTEN, PI3K, and AKT1 expressions/phosphorylations. Open circles indicate each individual value. Horizontal bars indicate mean value. Error bars indicate SEM (*, # *p* < 0.05 vs. control- and vehicle (Veh)-treated epileptic rats, respectively; one-way ANOVA with post hoc Bonferroni’s multiple comparison; *n* = 7, respectively).

In the present study, perampanel and GYKI 52,466 inhibited PI3K/AKT1-mediated GRIA1 surface expression by upregulation of PTEN expression. Thus, we directly confirmed the role of PTEN in their anti-epileptic effects by co-treatment of BpV(pic), a PTEN inhibitor. BpV(pic) co-treatment increased spontaneous seizure activity in perampanel and GYKI 52466-treated epileptic rats (*p* < 0.05 vs. perampanel and GYKI 52466, Mann–Whitney U-test and Student’s *t*-test, *n* = 7, respectively; Figure 7A and Figure S7). BpV(pic) co-treatment did not affect total GRIA1 expression and p65-Ser311 NF-κB phosphorylation ratio in perampanel and GYKI 52466-treated epileptic rats (Figure 7B,C and Figure S7). However, BpV(pic) co-treatment diminished PTEN expression to 0.73- and 0.78-fold of perampanel- and GYKI 52466-treated animal level, respectively (*p* < 0.05 vs. perampanel and GYKI 52466, one-way ANOVA with post hoc Bonferroni’s multiple comparison, *n* = 7, respectively; Figure 7B,C and Figure S7). BpV(pic) elevated PI3K phosphorylation ratio to 1.7- and 1.81-fold of perampanel- and GYKI 52466-treated animal level, respectively (*p* < 0.05 vs. perampanel and GYKI 52466, one-way ANOVA with post hoc Bonferroni’s multiple comparison, *n* = 7, respectively; Figure 7B,C and Figure S7). Following BpV(pic) treatment, AKT1 phosphorylation ratio was also increased to 2- and 1.78-fold of perampanel- and GYKI 52466-treated animal, respectively (*p* < 0.05 vs. perampanel and GYKI 52466, one-way ANOVA with post hoc Bonferroni’s multiple comparison, *n* = 7, respectively; Figure 7B,C and Figure S7). In addition, BpV(pic) increased Memb./Total GRIA1 ratio to 1.5- and 1.52-fold of perampanel- and GYKI 52466-treated animal, respectively (*p* < 0.05 vs. perampanel and GYKI 52466, one-way ANOVA with post hoc Bonferroni’s multiple comparison, *n* = 7, respectively; Figure 7B,C). Taken together, these findings indicate that in the epileptic hippocampus AMPAR hyperactivation may trigger a positive feedback loop via NF-κB/PTEN/PI3K/AKT1 signaling pathway, which would be involved in spontaneous seizure activity.

## 3. Discussion

**Figure 8:**
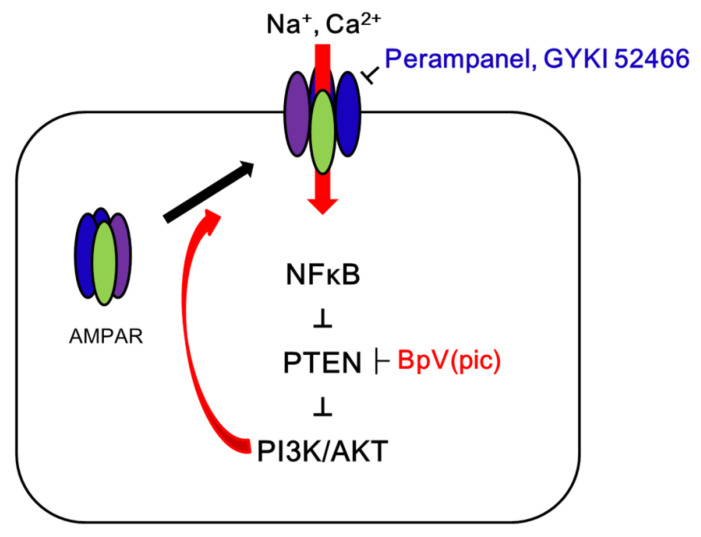
Scheme of the role of GRIA1 activation in epileptic rats. In the epilepsy hippocampus, α-amino-3-hydroxy-5-methyl-4-isoxazolepropionic acid receptor (AMPAR) hyperactivation leads to spontaneous seizure activity, which results in NF-κB-mediated PTEN downregulation. Subsequently, the reduced PTEN expression deregulates PI3K/AKT1 pathway that facilitates GRIA1 trafficking. Blockade of AMPAR by perampanel or GYKI 52,466 inhibits this signal cascade, which is abrogated by BpV(pic). Therefore, the present study demonstrates that PTEN may be required for the anti-epileptic effects of AMPAR antagonists.

In the present study, we found that blockade of AMPAR upregulated PTEN expression, which inhibited surface GRIA1 expression in the epileptic hippocampus (Figure 8).

Since AMPAR is essential for regulating the strength of excitatory neurotransmission, AMPAR seems to be a critical factor in the generation and spread of epileptic activity [4,5]. However, expression level of AMPAR subunits are decreased in the epileptic hippocampus of animal models [6]. Furthermore, the concentration of the whole hippocampal synaptosomal GRIA1 is reduced in chronic epilepsy rats [18]. Therefore, it has been still elusive how AMPAR is involved in the seizure onset and neuronal hyperexcitability in the epileptic hippocampus. In the present study, we found that total- and membrane GRIA1 expression levels were lower in the epileptic hippocampus than those in control one. However, Memb./Total GRIA1 ratio in the epileptic hippocampus was ≈1.5-fold higher than that in control hippocampus. These findings indicate that the increased surface GRIA1 expression/trafficking may be involved in the ictogenesis in epileptic rats, in spite of the reduced total GRIA1 expression. In addition, the present data demonstrate that both perampanel and GYKI 52,466 reduced total GRIA1 expression and Memb./Total GRIA1 ratio in the epileptic hippocampus, accompanied by reducing spontaneous seizure activity. These findings indicate that the anti-epileptic effect of blockade of AMPAR may be relevant to inhibitions of GRIA1 trafficking as well as its total expression in the epileptic hippocampus.

The PI3K/AKT1 signaling pathway is activated in epilepsy animal models and in patients who have temporal lobe epilepsy (TLE) with hippocampal sclerosis [25]. PI3K/AKT1 activation promotes seizure activity [26]. In addition, hyperactive PI3K/AKT1 signaling pathways play a role in seizure-induced memory deficits as well as aberrant spine morphology [27,28]. Consistent with these previous reports, the present study suggests that in epileptic hippocampus total PI3K/AKT1 phosphorylations were higher than those in the control hippocampus. In addition, immunostaining revealed that AKT1 phosphorylation was enhanced in dentate granule cells and neuroglial cells, as compared to the control hippocampus. Regarding the remaining GRIA1 expression in dentate granule cells of the epileptic hippocampus in the present study, it is likely that the upregulated PI3K/AKT1 activities may be relevant to AMPAR-mediated excitability in the epileptic hippocampus. Indeed, both perampanel and GYKI 52,466 reduced PI3K/AKT1 phosphorylation ratios to control levels indicating that AMPAR activation would increase PI3K/AKT1 activities. Therefore, our findings suggest that PI3K/AKT1 activation may play an important role in the increased Memb./Total GRIA1 ratio in the epileptic hippocampus.

Mutation or inactivation of PTEN contributes to seizures in human patients and animal models [29,30,31]. In addition, PTEN inhibition promotes GRIA1 trafficking to hippocampal synapses [17]. In the present study, PTEN expression level was lower in the epileptic hippocampus than that in the control one, accompanied by the increases in PI3K/AKT1 activities as well as Memb./Total GRIA1 ratios. Both perampanel and GYKI 52,466 restored PTEN expression in epileptic rats. Furthermore, BpV(pic) abrogated the anti-epileptic effects of perampanel and GYKI 52,466 in epileptic rats concomitant with the rebound of PI3K/AKT1 phosphorylations and Memb./Total GRIA1 ratios. Considering that PI3K that is negatively regulated by PTEN is required for maintaining AMPAR clustering at the postsynaptic membranes and its surface expression [12], our findings indicate that the downregulation of PTEN may trigger PI3K/AKT1-mediated GRIA1 trafficking in the epileptic hippocampus.

It is well known that NF-κB is sensitive to Ca^2+^ signals, since nuclear p65 NF-κB translocation is activated in response to the elevated intracellular Ca^2+^ level via N-methyl-D-aspartate (NMDA) receptor, AMPAR, and L-type voltage-gated Ca^2+^ channel [32,33]. The increased intracellular Ca^2+^ level activates calcium-calmodulin kinase II (CaMKII) in hippocampal neurons, which subsequently activates NF-κB [34]. However, we have reported that perampanel increases CAMKII activity (phosphorylation) in the hippocampus of chronic epileptic rats [9]. Thus, it is unlikely that AMPAR would activate NF-κB mediated by CAMKII. On the other hand, Ca^2+^ influx itself through activations of ionotropic glutamate receptors is sufficient to activate NF-κB through the canonical pathway [34,35,36]. Under unstimulated condition, NF-κB is bound to inhibitor of kB (IκB) in the cytoplasm. In response to stimuli, the elevated intracellular Ca^2+^ level activates IκB kinase complex (IKK) that phosphorylates and degrades IκB, and subsequently NF-κB is released from IκB [34]. Thus, it is likely that AMPAR activates the NF-κB signaling pathway through the canonical pathway in the hippocampus of chronic epileptic rats in the present study.

NF-κB-mediated signaling pathway positively regulates AKT1 activity [37,38]. In previous studies, we have reported that status epilepticus (SE, a prolonged seizure activity) elevates p65-S311 NF-κB phosphorylation only in dentate granule cells, but not CA1 and CA3 neurons [22,23]. Consistent with these previous reports, the present data reveal that p65-S311 NF-κB phosphorylation ratio was enhanced in dentate granule cells of epileptic rats. In addition, both perampanel and GYKI 52,466 diminished p65-S311 NF-κB phosphorylation ratio, as compared to vehicle. p65-S311 NF-κB phosphorylation enhances its interaction with cAMP response element-binding protein (CREB)-binding protein (CBP), which is a hallmark of NF-κB function to activate NF-κB-dependent transcription [21]. Interestingly, NF-κB negatively regulates PTEN expression through sequestration of limiting pools of the transcriptional co-activator CBP/p300, independent of p65 NF-κB DNA binding or transcription function. In addition, restoration of PTEN expression inhibits NF-κB transcriptional activity, indicating a negative regulatory loop involving PTEN and NF-κB [24,39]. Since PTEN inhibition did not affect p65-S311 NF-κB phosphorylation ratio reduced by perampanel or GYKI 52,466 in the epileptic hippocampus, our findings indicate that seizure-induced NF-κB activation may abrogate PTEN expression in the epileptic hippocampus.

Although no literature describes the effect of anti-epileptic drugs acting as Na^+^ blockers (lacosamide, lamotrigine, and carbamazepine) on GRIA1 surface expression, the chronic treatment of valproic acid (VPA, a Na^+^ blocker as well as γ-aminobutyric acid (GABA) enhancer) for 4 weeks reduces surface GRIA1 expression without altering total GRIA1 expression, which is mediated by attenuating PKA-mediated GRIA1-S845 phosphorylation [40]. However, perampanel increases GRIA1-S845 phosphorylation and PKA activity in the hippocampus of chronic epileptic rats, while it reduces total GRIA1 expression [9]. Thus, it is likely that the enhanced GRIA1 phosphorylations may be one of the adaptive responses for the diminished GRIA1 expression or AMPAR-mediated currents in the hippocampus of epileptic rats [9]. With respect to these previous reports, it is plausible that AMPAR antagonists may reduce surface GRIA1 expression, independent of GRIA1 phosphorylations.

In normal neurons, more than 95% of AMPARs contain the GRIA2 subunit, thus most AMPARs are Na^+^-permeable and Ca^2+^-impermeable. Since GRIA2-lacking AMPAR is Ca^2+^-permeable, the formation of GRIA2-lacking AMPARs enhances the toxicity of endogenous and exogenous glutamate [41]. Indeed, heterozygous de novo *GRIA2* mutations cause intellectual disability and neurodevelopmental abnormalities including autism spectrum disorder, Rett syndrome-like features, and seizures or developmental epileptic encephalopathy [42]. Furthermore, GRIA2 surface expression is decreased in the hippocampus of epileptic animals, as compared to normal animals [43,44], and GYKI-52466 effectively reduces intracellular Ca^2+^ level by inhibiting GRIA2-lacking AMPARs [45]. In the present study, Memb. GRIA1/GRIA2 ratio was higher in the epileptic animals than that in control animals. Furthermore, both perampanel and GYKI-52466 reduced the surface expression of GRIA1, but not GRIA2. These findings suggest the possibility that the increased GRIA2-lacking AMPAR population may be also involved in the ictogenesis in the epileptic hippocampus. In addition, the anti-epileptic effects of AMPAR antagonists may be relevant to the reduced the formation of GRIA1-homo or GRIA2-lacking AMPARs by inhibiting GRIA1 trafficking.

Recently, it has been reported that p97/valosin-containing protein (VCP), a GRIA1 interacting protein, specifically interacts with the GRIA1 subunit of homomeric AMPARs, but not the GRIA1 subunit that is heteromerized with the GRIA2 subunit. In addition, p97/VCP promotes the formation of GRIA1-homo AMPARs, but retains the homomeric receptors intracellularly under basal conditions and rapidly releases them into the postsynaptic membrane after induction of long-term potentiation (LTP) [46]. Considering the role of GRIA2-lacking AMPARs in epilepsy aforementioned [43,44,45], it is plausible that p97/VCP may play a role in the ictogenesis by promoting the formation of GRIA2-lacking AMPARs. Furthermore, p97/VCP regulates NF-κB activation, since p97/VCP is essentially required for efficient liberation of p65 NF-κB from IκB, preceding p97/VCP-promoted timely and efficient degradation of IκB as well as simultaneous p65 NF-κB nuclear translocation [47]. Thus, it is also presumable that p97/VCP may be involved in regulating surface GRIA1 expression and AMPAR-mediated NF-κB activation in the hippocampus of epileptic rats.

On the other hand, p97/growth factor receptor bound protein 2-associated binder 2 (Gab2) is a member of a large family of scaffold proteins that play essential roles in signal transduction. Gab2 becomes tyrosine-phosphorylated in response to a variety of growth factors and forms multimolecular complexes with SH2 domain-containing signaling molecules [48]. Indeed, p97/Gab2 is required for the PI3K/AKT1 signaling pathway [49] and p97/Gab2-mediated PI3K/AKT1 activation is involved in the pathogenesis of Alzheimer’s disease (AD) [50] that shows the reduced GRIA1 surface expression [51]. However, p97/Gab2 protein expression is remarkably reduced in the hippocampus of TLE patients and chronic epileptic rats [52]. In addition, the present data reveal the upregulations of GRIA1 surface expression and PI3K phosphorylation in the epileptic hippocampus. Thus, it is unlikely that p97/Gab2 may be involved in the PI3K/AKT1 signaling pathway in the epileptic hippocampus, which would be abrogated by AMPAR antagonists.

## 4. Materials and Methods

### 4.1. Experimental Animals and Chemicals

In the present study, we used adult male Sprague-Dawley (SD) rats (7 weeks old). Animals were in-housed under controlled conditions (22 ± 2 °C, humidity 55% ± 5%, a light-dark cycle on a 12-h on-off cycle) with ad libitum access to water and food throughout the experiments. All experimental protocols described below were approved by the Institutional Animal Care and Use Committee of Hallym University (Hallym 2018-2, 26 Apr 2018). Every effort was made to reduce the number of animals employed and to minimize animal discomfort. All reagents were obtained from Sigma-Aldrich (St. Louis, MO, USA), except as noted.

### 4.2. Generation of Epileptic Rats

Rats were given LiCl (127 mg/kg, i.p.) 24 h before the pilocarpine treatment. Animals were treated with pilocarpine (30 mg/kg, i.p.) 20 min after atropine methylbromide (5 mg/kg i.p.). Two hours after SE onset, diazepam (Valium; Hoffmann-la Roche, Neuilly-sur-Seine, France; 10 mg/kg, i.p.) was administered to terminate convulsive SE and repeated, as needed, although it could not exclude the possibility that non-convulsive seizures would persist beyond the time of treatment. Control animals received saline in place of pilocarpine. Animals were video monitored 8 h a day for 4 weeks for selecting chronic epileptic rats showing spontaneous recurrent seizures [9,53]. Behavioral seizure severity was evaluated according to Racine’s scale: 1, immobility, eye closure, twitching of vibrissae, sniffing, facial clonus; 2, head nodding associated with more severe facial clonus; 3, clonus of one forelimb; 4, rearing, often accompanied by bilateral forelimb clonus; and 5, rearing with loss of balance and falling accompanied by generalized clonic seizures. We classified epileptic rats that showed behavioral seizure activities with seizure score ≥ 3 more than once.

### 4.3. Electrode Implantation

Control and epileptic rats (4 weeks after SE) were implanted with monopolar stainless steel electrodes (Plastics One, Roanoke, VA, USA) in the right hippocampus under Isoflurane anesthesia (3% induction, 1.5–2% for surgery, and 1.5% maintenance in a 65:35 mixture of N_2_O:O_2_) using the following coordinates: −3.8 mm posterior; 2.0 mm lateral; −2.6 mm depth. Throughout surgery, core temperature of each rat was maintained at 37–38 °C. Electrode was secured to the exposed skull with dental acrylic.

### 4.4. Drug Trials and Quantification of Seizure Activity

Three days after electrode implantation, baseline seizure activity was determined over 3 days. Thereafter, perampanel (2-(2-oxo-1-phenyl-5-pyridin-2-yl-1,2-dihydropyridin-3-yl)benzonitrile, a non-competitive AMPAR antagonist; 8 mg/kg, i.p, Eisai Korea Inc., Seoul, South Korea), GYKI 52,466 (a non-competitive AMPAR inhibitor, 10 mg/kg, i.p.) or saline (vehicle) was daily administered at a certain time of the day (PM 6:00) over a 1-week period [9,54]. On the basis of previous studies [55,56], some animals were also given BpV(pic) (2 mg/kg, i.p.) with perampanel or GYKI 52466. EEG was recorded 2 h a day at the same time over a 1-week period. EEG signals were recorded with a DAM 80 differential amplifier (0.1–3000 Hz bandpass; World Precision Instruments, Sarasota, FL, United States) and the data were digitized (1000 Hz) and analyzed using LabChart Pro v7 (ADInstruments, Bella Vista, New South Wales, Australia). Behavioral seizure severity was also evaluated as aforementioned. After recording (18 h after the last treatment), animals were used for Western blot and immunohistochemical study.

### 4.5. Membrane Fraction and Western Blots

A total of 18 h after the last compound treatment, animals were sacrificed by decapitation, and the hippocampi were obtained. The hippocampal tissues were homogenized, and protein concentration determined using a Micro BCA Protein Assay Kit (Pierce Chemical, Rockford, IL, USA). To analyze membrane GRIA1 expression, we used a subcellular Protein Fractionation Kit for Tissues (Thermo Scientific, USA), according to the manufacturer’s instructions. Western blot was performed by the standard protocol. The primary antibodies used in the present study are listed in Table 1. The bands were detected and quantified on an ImageQuant LAS4000 system (GE Healthcare Korea, Seoul, South Korea). The values of each sample were normalized with the corresponding amount of β-actin or N-cadherin. The ratio of phosphoprotein to total protein was described as phosphorylation level.

### 4.6. Tissue Processing and Immunohistochemistry

Under urethane anesthesia (1.5 g/kg, i.p.), animals were transcardially perfused with 0.9% saline followed by 4% paraformaldehyde in 0.1 M phosphate buffer (PB, pH 7.4). After perfusion, the brains were extracted, post-fixed in the same fixative overnight, and then stored in 30% sucrose/0.1 M PBS. Coronal sections were sliced at a 30-μm thickness with a freezing microtome. Then the sections were washed three times using PBS (0.1 M, pH 7.3). After that, sections were incubated in 0.1% bovine serum albumin and subsequently primary antibody (Table 1). Tissue sections visualized with appropriate Cy2-conjugated secondary antibodies. Immunoreaction was observed using an Axio Scope microscope (Carl Zeiss Korea, Seoul, South Korea). To establish the specificity of the immunostaining, a negative control test was carried out with preimmune serum instead of the primary antibody. No immunoreactivity was observed for the negative control in any structures. All experimental procedures in this study were performed under the same conditions and in parallel. Some sections were used for Cresyl violet staining to confirm epileptic animals and neuronal death.

### 4.7. Data Analysis

Comparisons of Western blot data among groups were performed using one-way ANOVA followed by Bonferroni’s post hoc comparisons after evaluating the values on normality using Shapiro–Wilk *W*-test. Mann–Whitney U-test (seizure frequency and seizure severity), Student’s *t*-test (comparison of seizure duration), and *χ*^2^ test (comparison of seizure frequency between responder and non-responder) were also used to determine statistical significance of data. A *p*-value less than 0.05 was considered to be significant.

## 5. Conclusions

The present study demonstrates that PTEN is pivotal for anti-epileptic effects of AMPAR antagonists in the epileptic hippocampus, which regulates surface AMPAR expression (Figure 8). Thus, our findings provide the experimental evidence suggesting that PTEN activation may be one of the therapeutic strategies for better outcomes of epilepsy in clinical studies.

## Figures and Tables

**Table 1:** Primary antibodies used in the present study.

Antigen	Host	Manufacturer (Catalog Number)	Dilution Used
AKT1	Rabbit	Cell signaling (#9272)	1:1000 (WB)
			1:100 (IH)
GRIA1	Mouse	Synaptic systems (182011)	1:1000 (WB)
GRIA2		Sigma (AB1768-I)	1:1000 (WB)
N-cadherin	Rabbit	Abcam (ab18203)	1:4000 (WB)
NF-κB RelA p65	Rabbit	Abcam (ab16502)	1:1000 (WB)
			1:200 (IH)
p-AKT1-S473	Rabbit	Cell signaling (#4060)	1:1000 (WB)
			1:100 (IH)
p-NF-κB RelA p65-S311	Rabbit	Abcam (ab194926)	1:2000 (WB)
p-PI3K-Y458	Rabbit	Cell signaling (#4228S)	1:1000 (WB)
p-PTEN-S380/Y382/Y383	Rabbit	Cell signaling (#9549)	1:1000 (WB)
PI3K	Rabbit	Cell signaling (#4292S)	1:1000 (WB)
			1:200 (IH)
PTEN	Rabbit	Abcam (ab170941)	1:5000 (WB)
β-actin	Mouse	Sigma (#A5316)	1:5000 (WB)

IH: immunohistochemistry; WB: Western blot.

## Supplementary Materials

Supplementary Materials can be found at https://www.mdpi.com/1422-0067/21/16/5643/s1. Figure S1: Representative full-gel images of Western blots in Figure 1, Figure S2: Representative full-gel images of Western blots in Figure 2, Figure S3: Representative full-gel images of Western blots in Figure 3, Figure S4: Representative full-gel images of Western blots in Figure 4, Figure S5: Representative full-gel images of Western blots in Figure 5, Figure S6: Representative full-gel images of Western blots in Figure 6, and Figure S7: Representative full-gel images of Western blots in Figure 7.

## Abbreviations

AMPAR	α-Amino-3-hydroxy-5-methyl-4-isoxazolepropionic acid receptor
BpV(pic)	Dipotassium bisperoxovanadium(pic) dihydrate
NF-κB	Nuclear factor-kappaB
PI3K	Phosphoinositide 3-kinase
PTEN	Phosphatase and tensin homolog deleted on chromosome 10
