# Supplementary material for: PTEN Is Required for The Anti-Epileptic Effects of AMPA Receptor Antagonists in Chronic Epileptic Rats
Source: Int J Mol Sci. 2020 Aug 6;21(16):5643. doi: 10.3390/ijms21165643 (PMC7460838; doi:10.3390/ijms21165643)
Supplement: Supplementary file 1 [file ijms-21-05643-s001.pdf]

Supplementary Informations

PTEN is required for the anti-epileptic effects of AMPA receptor antagonists in chronic epileptic rats

Ji-Eun Kim[1,2], Hana Park[1,2], Ji-Eun Lee[1,2], Tae-Hyun Kim[1,2], Tae-Cheon Kang[1,2*]

[1]Department of Anatomy and Neurobiology, College of Medicine, Hallym University, Chuncheon 24252, South Korea

[2]Institute of Epilepsy Research, College of Medicine, Hallym University, Chuncheon 24252, South Korea

*Correspondence to: T. -C. Kang, Department of Anatomy and Neurobiology, College of Medicine, Hallym University, Chuncheon, Kangwon-Do 24252, South Korea; Tel: +82-33-248-2524; Fax: +82-33-248-2525; E-mail: tckang@hallym.ac.kr

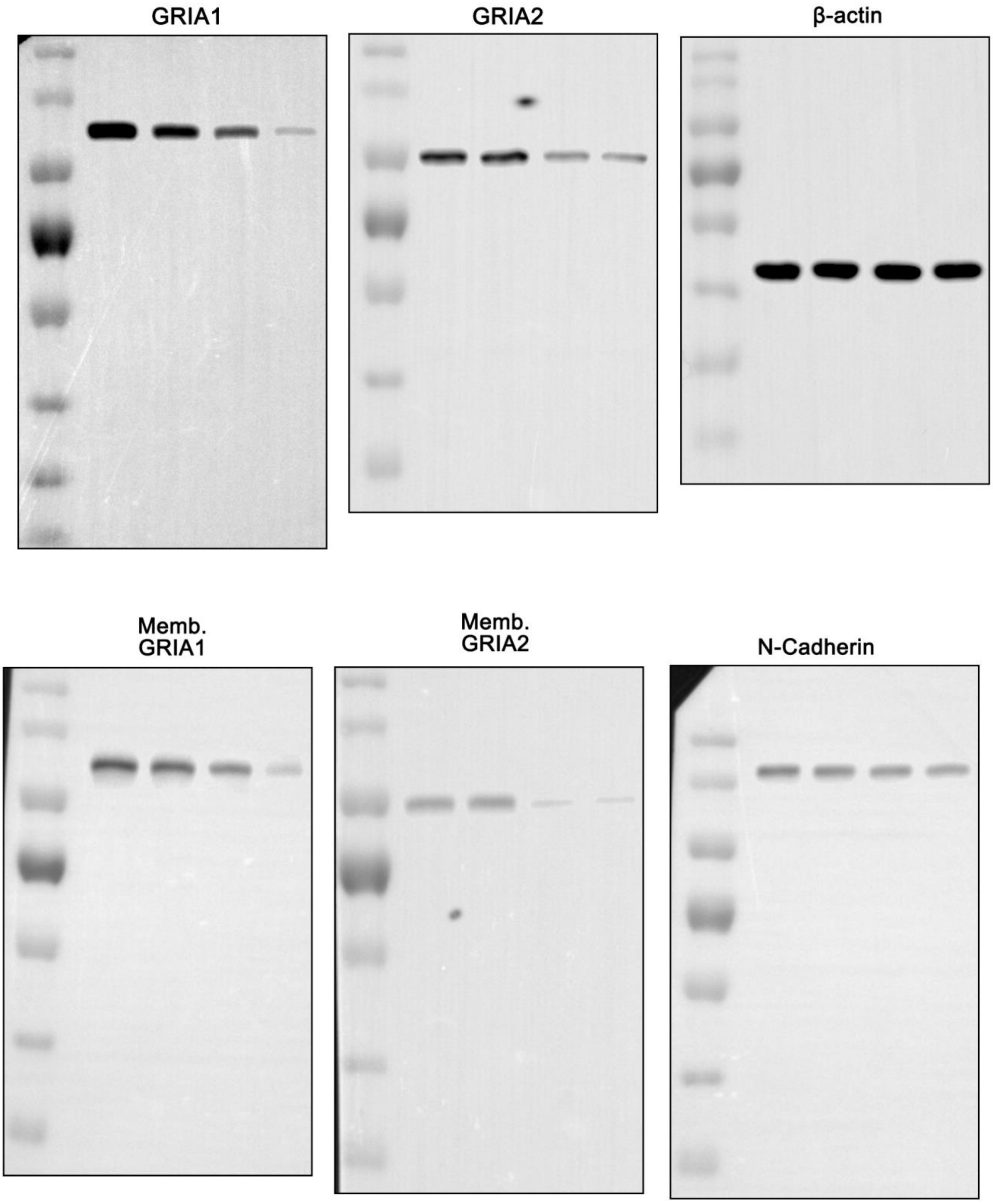

Supplementary Figure 1. The whole gel images of Western blot in Figure 1.

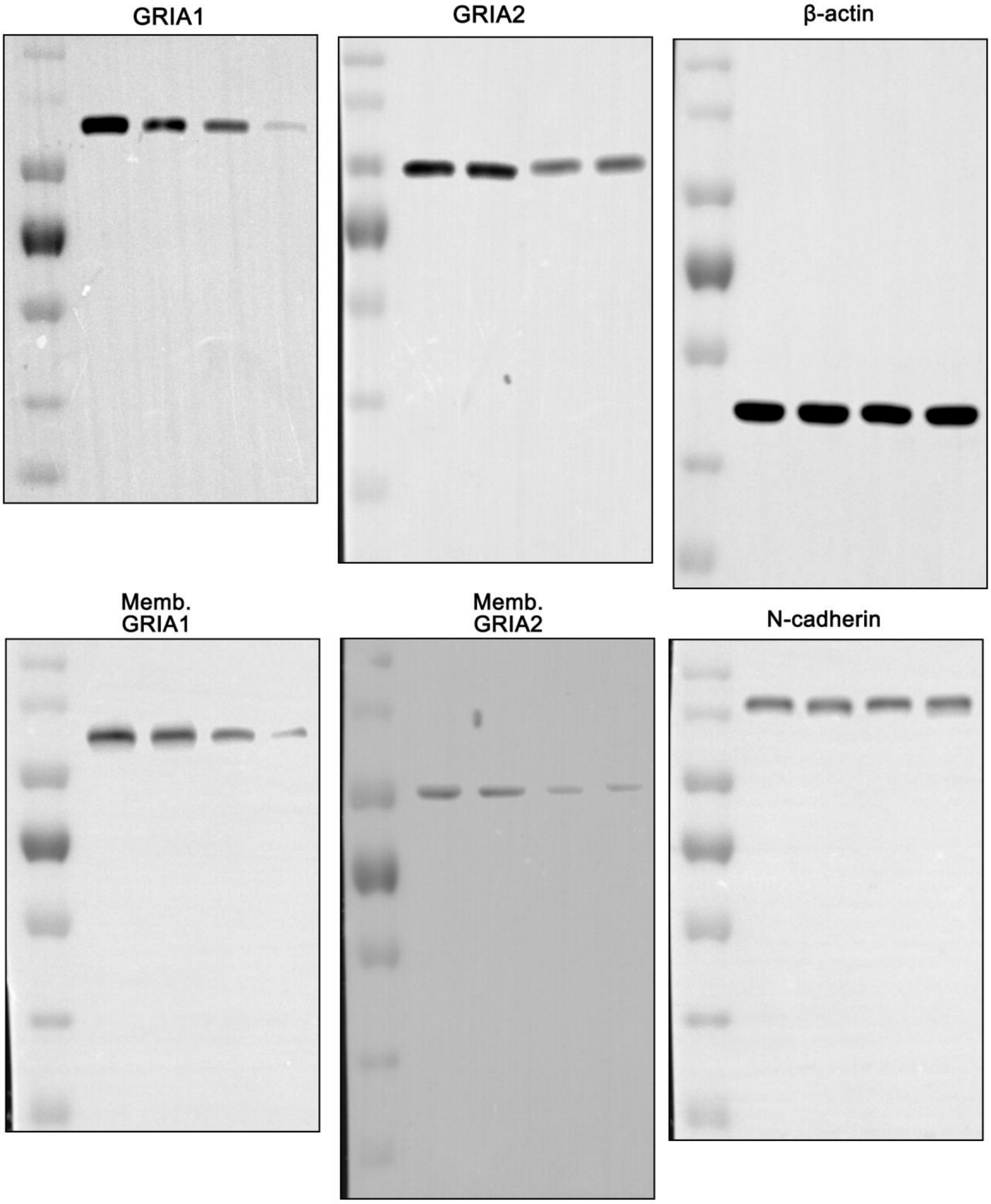

Supplementary Figure 2. The whole gel images of Western blot in Figure 2.

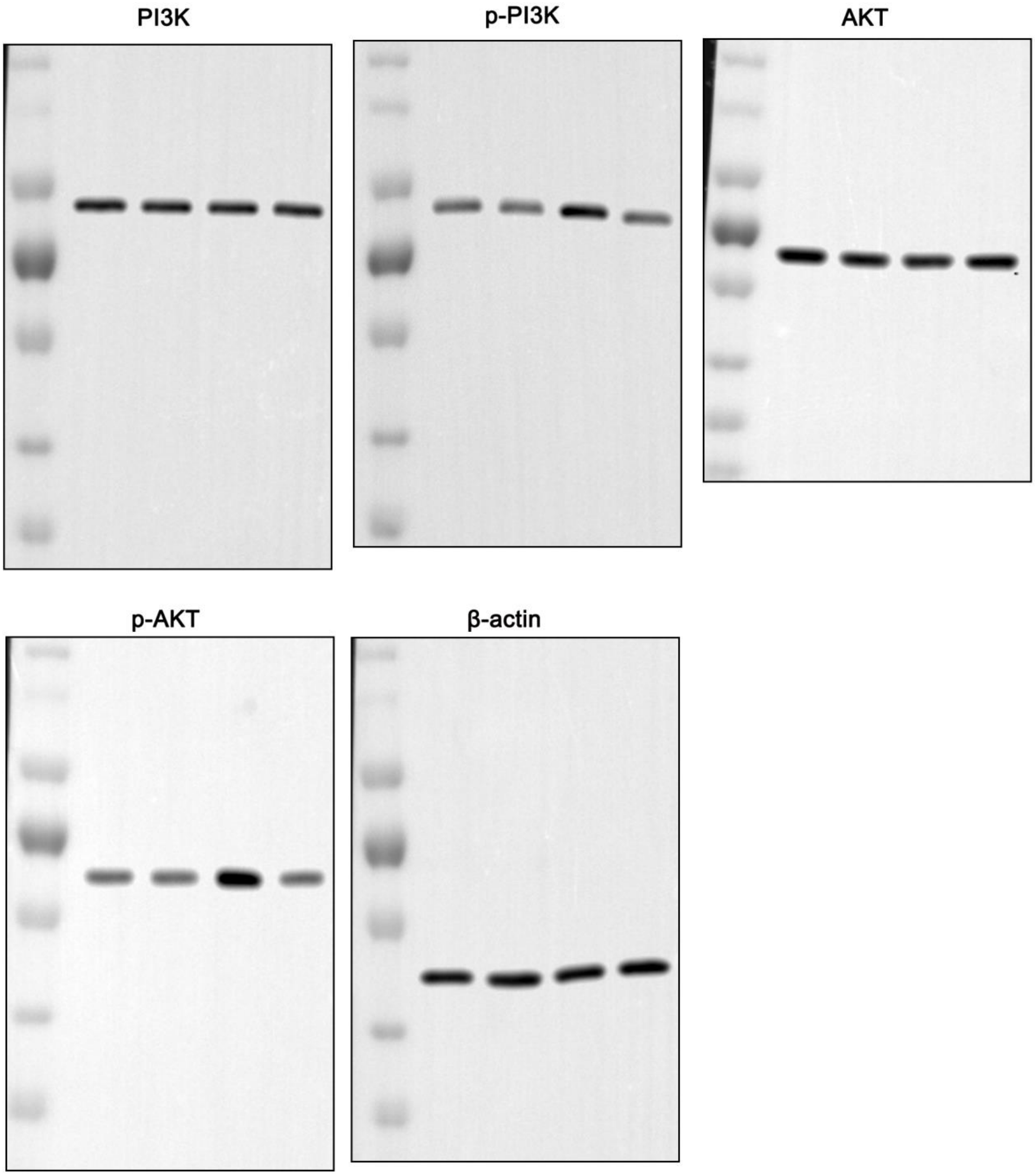

Supplementary Figure 3. The whole gel images of Western blot in Figure 3.

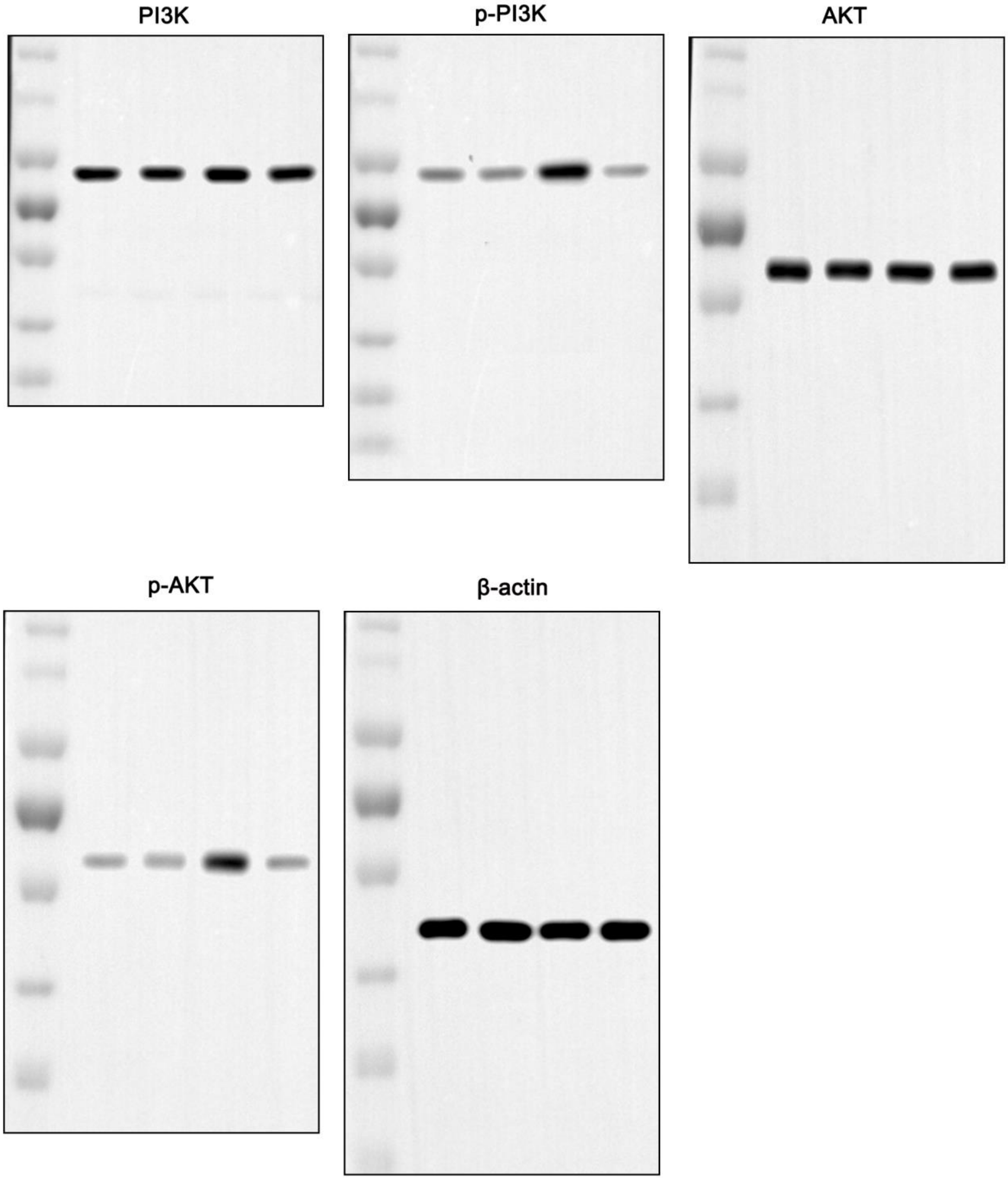

Supplementary Figure 4. The whole gel images of Western blot in Figure 4.

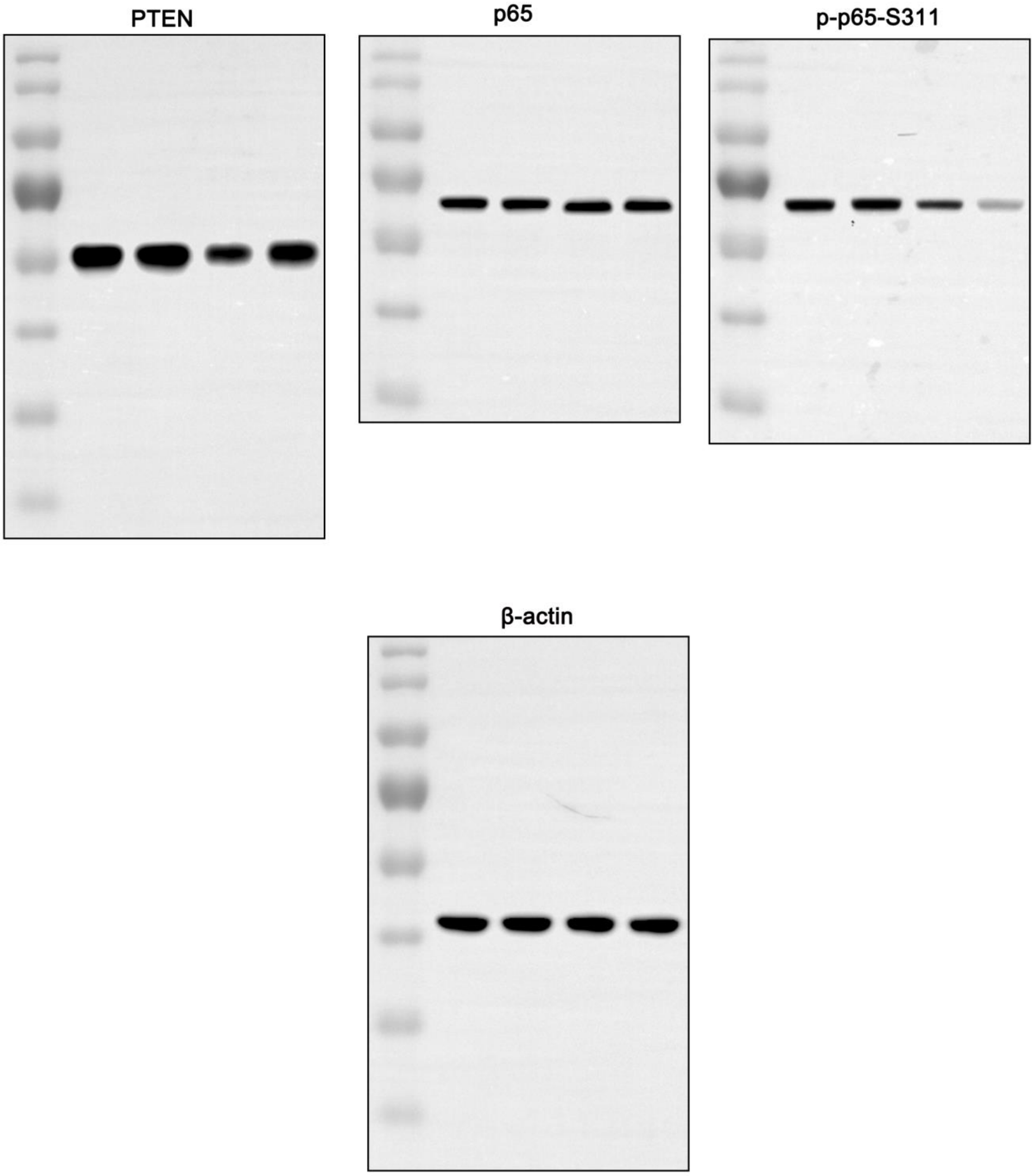

Supplementary Figure 5. The whole gel images of Western blot in Figure 5.

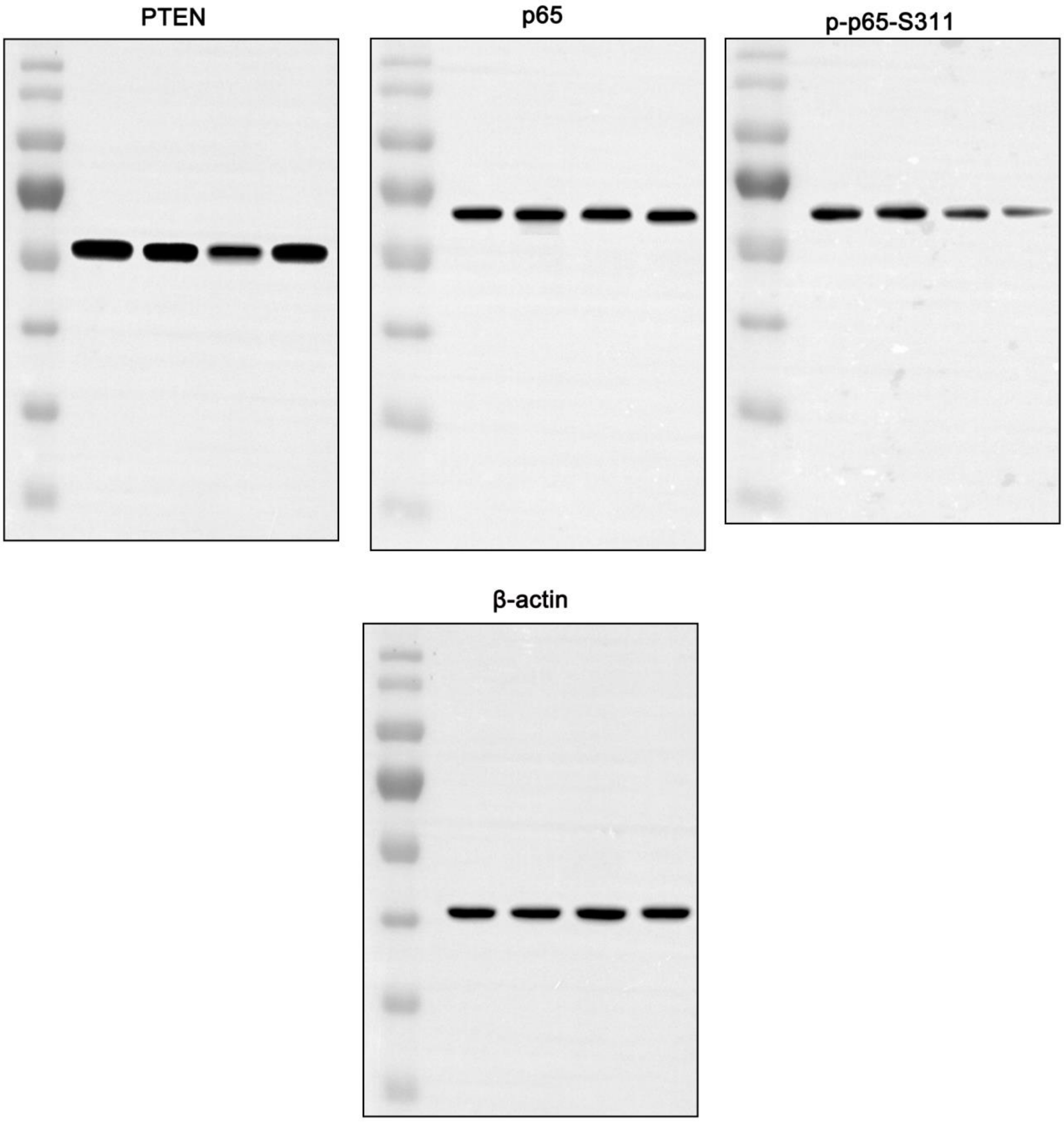

Supplementary Figure 6. The whole gel images of Western blot in Figure 6.

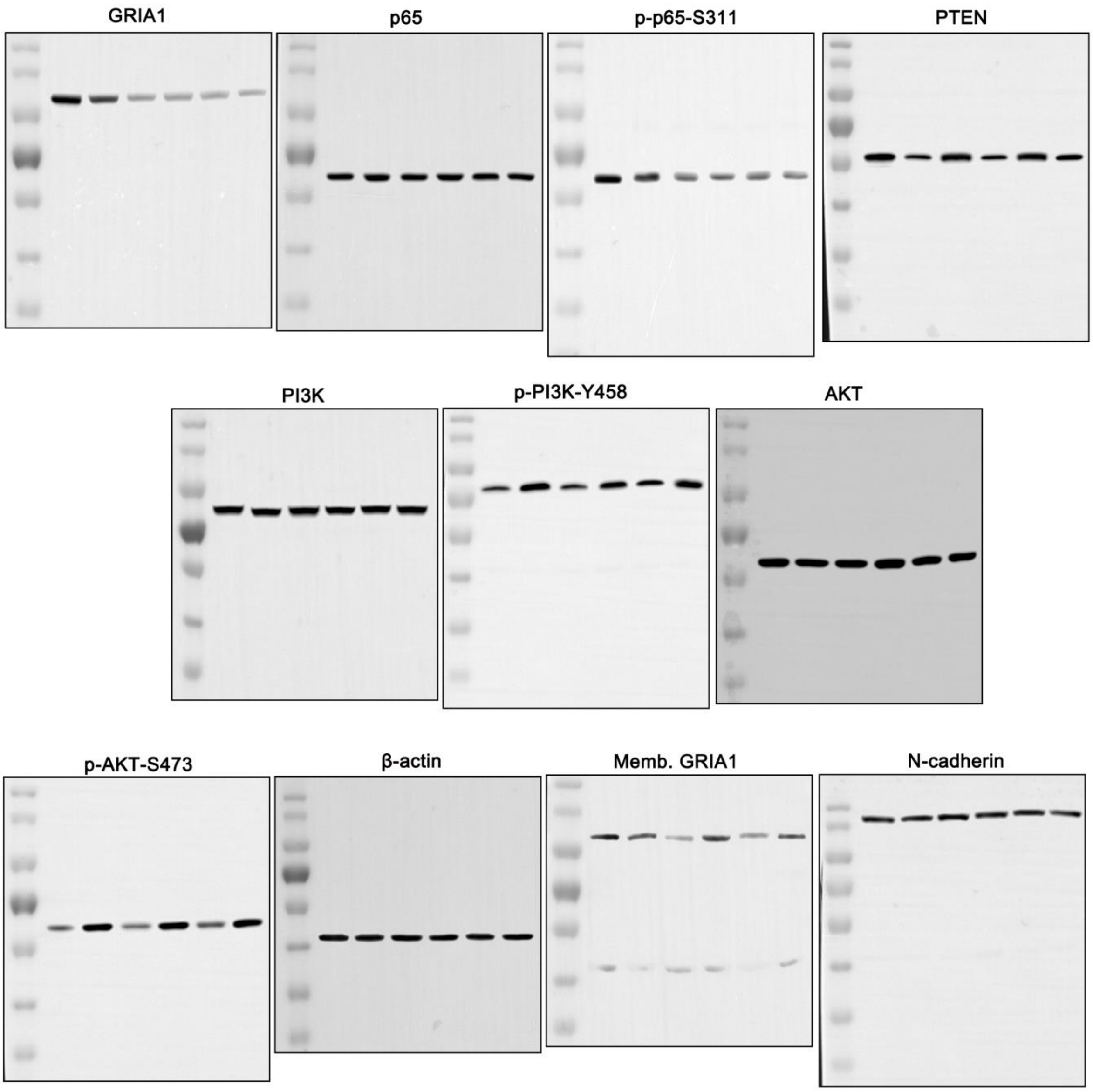

Supplementary Figure 7. The whole gel images of Western blot in Figure 7.
